# Echinacoside Increases Sperm Quantity in Rats by Targeting the Hypothalamic Androgen Receptor

**DOI:** 10.1038/s41598-018-22211-1

**Published:** 2018-03-01

**Authors:** Zhihui Jiang, Bo Zhou, Xinping Li, Gordon M. Kirby, Xiaoying Zhang

**Affiliations:** 1 Research Center of Modern Biotechnology, School of Biotechnology and Food Engineering, Anyang Institute of Technology, Anyang, Henan 455000 China; 2 College of Veterinary Medicine, Northwest A & F University, Yangling, Shaanxi 712100 China; 3 Department of Biomedical Sciences, Ontario Veterinary College, University of Guelph, Guelph, Ontario N1G 2W1 Canada

## Abstract

Male infertility is a major health issue with an estimated prevalence of 4.2% of male infertility worldwide. Our early work demonstrated that *Cistanche* extracts protect against sperm damage in mice and that echinacoside (ECH) is one of the major active components. Here we report an essential role for ECH, a natural product that reverses or protects against oligoasthenospermia in rats. ECH was assayed by HPLC, the quantity and quality of sperm was evaluated and hormone levels were determined by radioimmunosorbent assay. ECH reduced levels of androgen receptor (AR) and key steroidogenic-related genes as determined by Western blot and qPCR analysis. The interaction between ECH and AR were evaluated by indirect ELISA and molecular docking. The results show that ECH combined with hypothalamic AR in the pocket of Met-894 and Val-713 to inhibit transfer of AR from the cytoplasm to nuclei in the hypothalamus. While negative feedback of sex hormone regulation was inhibited, positive feedback was stimulated to increase the secretion of luteinizing hormone and testosterone subsequently enhancing the quantity of sperm. Taken together, these data demonstrate that ECH blocks AR activity in the hypothalamus to increase the quantity of sperm and protect against oligoasthenospermia in rats.

## Introduction

The production of testosterone in testicular cells is strongly regulated by the hypothalamic-pituitary-gonadal axis (HPG) by forming a homeostatic feedback loop^1^. Gonadotrophin releasing hormone (GnRH), secreted by the hypothalamus, can stimulate the secretion of luteinizing hormone (LH) from the pituitary gland, which further stimulates testosterone production in testicular Leydig cells. Testosterone is biosynthesized by a series of steroidogenic enzymes. As one of the major pathways, steroidogenic acute regulatory (StAR) protein can transport cholesterol from intracellular sources into the mitochondria^2^ where it is exposed to cholesterol side-chain cleavage enzyme (CYP11A1), 3β-hydroxysteroid dehydrogenase (HSD3β), 17α-hydroxylase (CYP17A1) and 17β-hydroxysteroid dehydrogenase (HSD 17β) that catalyze the conversion of cholesterol to testosterone^3,4^. Testosterone then negatively feeds back to the HPG element to down-regulate further LH secretion in a dose-dependent manner.

The effect of testosterone on the HPG axis feedback loop occurs by binding to the androgen receptor (AR), found in both the hypothalamus and the pituitary gland^5^. In the mice, ablation of the AR and minimal testosterone production causes levels of LH and follicle stimulating hormone (FSH) to increase^6^, suggesting that AR participates in the regulation of the negative feed loop. The classical genomic mechanism of testosterone signaling occurs when testosterone diffuses into the cell and binds to the AR. This ligand-receptor complex then translocates to the nucleus where it binds to androgen response elements (AREs) in the regulatory regions of testosterone-responsive genes to modify their translocation. Testosterone also induces the non-classical pathway of steroid hormone action, characterized by rapid events that lead to the activation of cytosolic signaling cascades normally triggered by growth factors^7,8^. Classical and non-classical testosterone pathways both contribute to maintaining spermatogenesis and fertility. However, the function of the AR is more important in the classical pathway as testosterone acts to increase sperm quality.

Echinacoside (ECH) is one of the bioactive components derived from the medicinal plant species of Echinacea^9^ and Cistanche^10^. With a broad spectrum of pharmacological activities, extracts of the Echinacea are one of the most popular herbal supplements in Europe and the US mainly due to their antioxidant properties^11^ and their ability to prevent the common cold^12^. Interestingly, *Cistanche* extracts and ECH have been traditionally used as a tonic agent to cure reproductive dysfunction and to boost male sexual activity in traditional Chinese medicine^10^. Some OTC products of *Cistanche* extraction have been developed as nourishing supplements and are gaining popularity in the health food markets of China and some other Asian countries (China Food and Drug Administration)^13^. However, the underlying mechanisms of ECH action remain unclear.

Male infertility is a major health issue with an estimated prevalence of 4.2% of male infertility worldwide^14^. The diagnosis of male infertility is currently based on the study of sperm quality including the analysis of seminal parameters such as sperm concentration, motility and morphology^15^. The estrogen-mimic Bisphenol A (BPA) is a widespread environmental contaminant that has been studied for its impact on male fertility in several species of animals and humans^16^. BPA disrupts the hypothalamic-pituitary-gonadal axis, inhibits the expressions of testicular steroidogenic enzymes and the synthesis of testosterone in the male pups^17^, causing a state of hypogonadotropic hypogonadism^18^. In this study, we investigate the effects of ECH on the sperm quality and hormone levels. In addition, BPA was chosen as a sperm injury model agent to further study the protective effect of ECH against poor sperm quality.

## Results

### ECH enhances the sperm quantity

The sperm numbers in cauda epididymis, sperm viability and sperm motility are presented in Table 1. Treatment with 80 mg/kg of ECH and 15 mg/kg of testosterone propionate (TP) significantly increased the epididymal sperm counts. However, there was no significant difference in sperm viability and sperm motility.

**Table 1:** Sperm numbers, sperm viability and motility in cauda epididymis.

Sperm parameters	Control	ECH_(L)_	ECH_(M)_	ECH_(H)_	TP
Sperm number in cauda epididymis (×10^7^)	10.5 ± 0.46^b^	12.3 ± 0.51^ab^	11.6 ± 0.62^b^	13.7 ± 0.44^a^	13.1 ± 0.36^a^
Sperm viability (%)	87.4 ± 6.95^a^	89.4 ± 4.54^a^	89.2 ± 8.10^a^	92.1 ± 6.85^a^	90.3 ± 6.32^a^
Sperm motility (%)	80.3 ± 8.52^a^	83.2 ± 5.16^a^	81.6 ± 12.2^a^	86.5 ± 10.1^a^	91.7 ± 6.25^a^

Note: ^a,b,c^Different letters represent groups that differ statistically (*p* < 0.05) based on ANOVA and post-hoc Tukey test.

### ECH increases testosterone and LH levels in serum, encephalon + pituitary and testis

**Figure 1:**
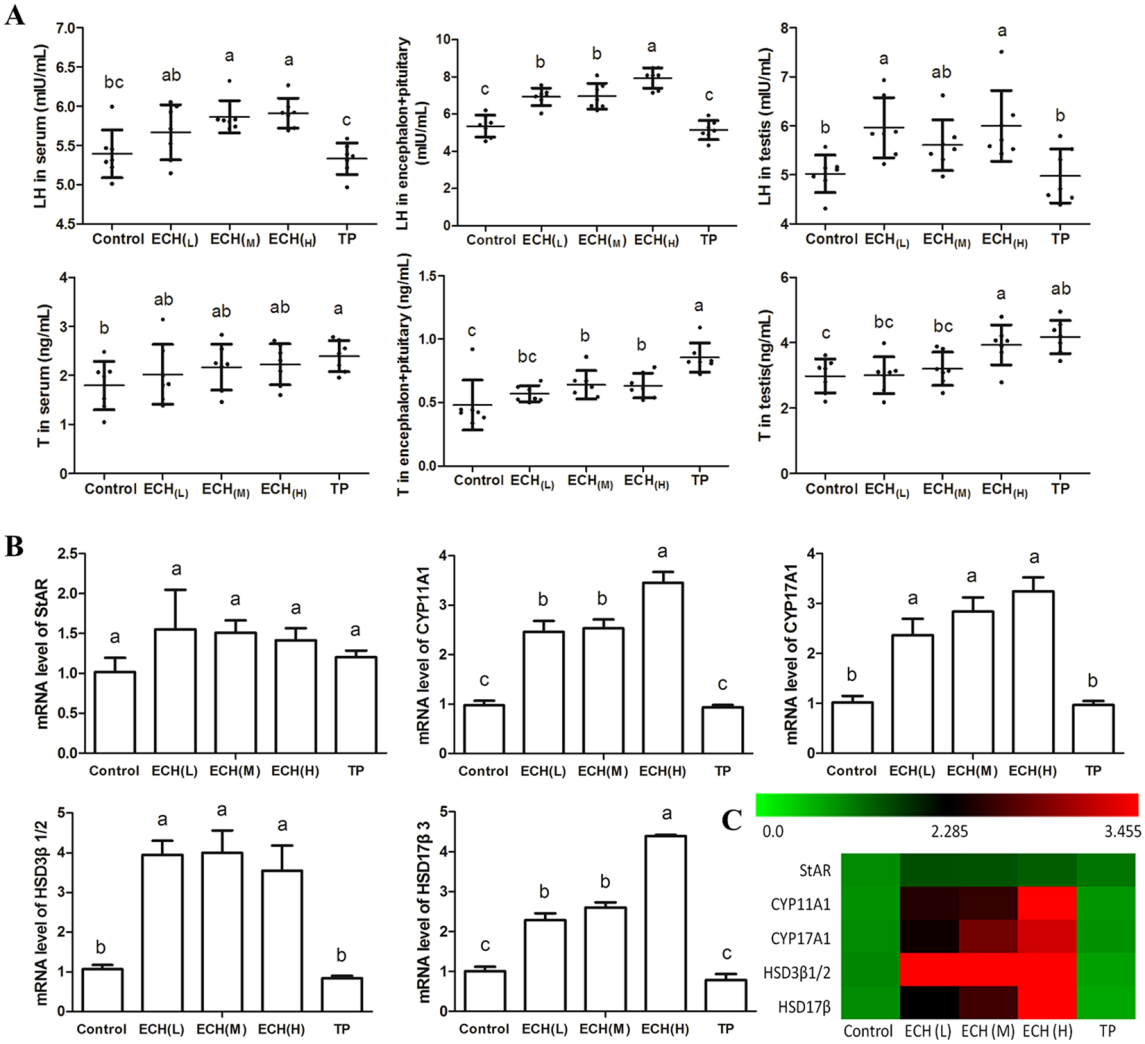
Effect of ECH on the levels of LH, testosterone and major steroidogenic enzymes. Note: (**A**) levels of LH in serum, encephalon + pituitary and testis (mIU/mL, mean ± SD); levels of T in serum, encephalon + pituitary and testis (ng/mL, mean ± SD); (**B**) expression of major steroidogenic enzymes; (**C**) hierarchical clustering of mRNA expression of different steroidogenic enzymes in testis. Green, green-red and red colors represent low-, medium- and high- abundance, respectively, n = 7. ^a,b,c^Different letters indicate statistically different groups (*p* < 0.05).

ECH treatment notably increased LH levels in the serum, encephalon + pituitary and testis (Fig. 1A). In the ECH_(H)_ group, LH levels significantly increased by 1.1-fold in serum, 1.4-fold in encephalon + pituitary and 1.2-fold in testis. Testosterone was significantly increased by 1.5 and 1.3-fold in encephalon + pituitary and testis, respectively, but not in serum after ECH_(H)_ treatment. After TP treatment, the testosterone levels were significantly increased by 1.6, 1.8 and 1.4-fold in serum, encephalon + pituitary and testis.

### ECH increased steroidogenic enzyme gene expression

ECH significantly increased the expressions of key steroidogenic enzymes in the testis (Fig. 1B). The mRNA levels of CYP11A1, CYP17A1, HSD3β1/2 and HSD17β in the ECH_(H)_ group were increased more than 3-fold, while the levels of StAR mRNA showed no significant differences with all ECH treatments (*p* > 0.05). TP did not significantly alter the expression of CYP11A1, CYP17A1, HSD3β1/2 and HSD17β. This is reflected in a comprehensive heatmap analysis of the effect of ECH on steroidogenic enzyme expression indicating that ECH significantly increased mRNA levels of CYP11A1, CYP17A1, HSD3β1/2 and HSD17β, and particularly HSD3β1/2 (Fig. 1C).

### ECH can cross the blood-brain barrier and to a lesser extent the blood-testis barrier

**Figure 2:**
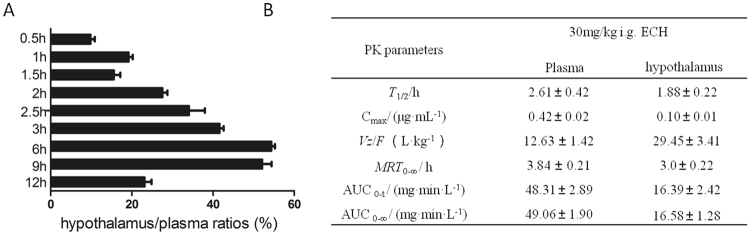
Distribution and pharmacokinetic parameters of ECH in the hypothalamus. Notes: (**A**) hypothalamus/plasma ratios (%) were calculated based on plasma and hypothalamus exposures of ECH (concentration) at 0.5 h, 1.0 h, 1.5 h, 2.0 h, 2.5 h, 3.0 h, 6.0 h, 9.0 h and 12.0 h after oral administration. (**B**) The pharmacokinetic (PK) parameters of ECH in plasma and hypothalamus. *T*_1/2_, half-life; C_max_, peak concentration; *Vz*/*F*, apparent volume of distribution; *MRT*, mean retention time; *AUC*, area under the concentration time curve.

To evaluate the androgen feedback loop at the HPG-axis, the ability of ECH to enter the hypothalamus was evaluated in a series of PK studies. ECH concentrations were detected in the hypothalamus after 12 h single oral administration of 30 mg/kg of ECH, and ECH showed marked hypothalamus penetrance with the mean hypothalamus/plasma ratio of 33.92% (Fig. 2A). The *T*_1/2_ values were 2.61 ± 0.42 h and 1.88 ± 0.22 h in plasma and the hypothalamus, respectively (Fig. 2B). However, very limited access of ECH into the testis was detected using HPLC. The concentrations of ECH in testis were 0.083 μg/mL, 0.043 μg/mL and 0.028 μg/mL at 0.5 h, 1 h and 1.5 h respectively, after treatment.

### ECH reduces hypothalamic AR translocation to the nucleus

**Figure 3:**
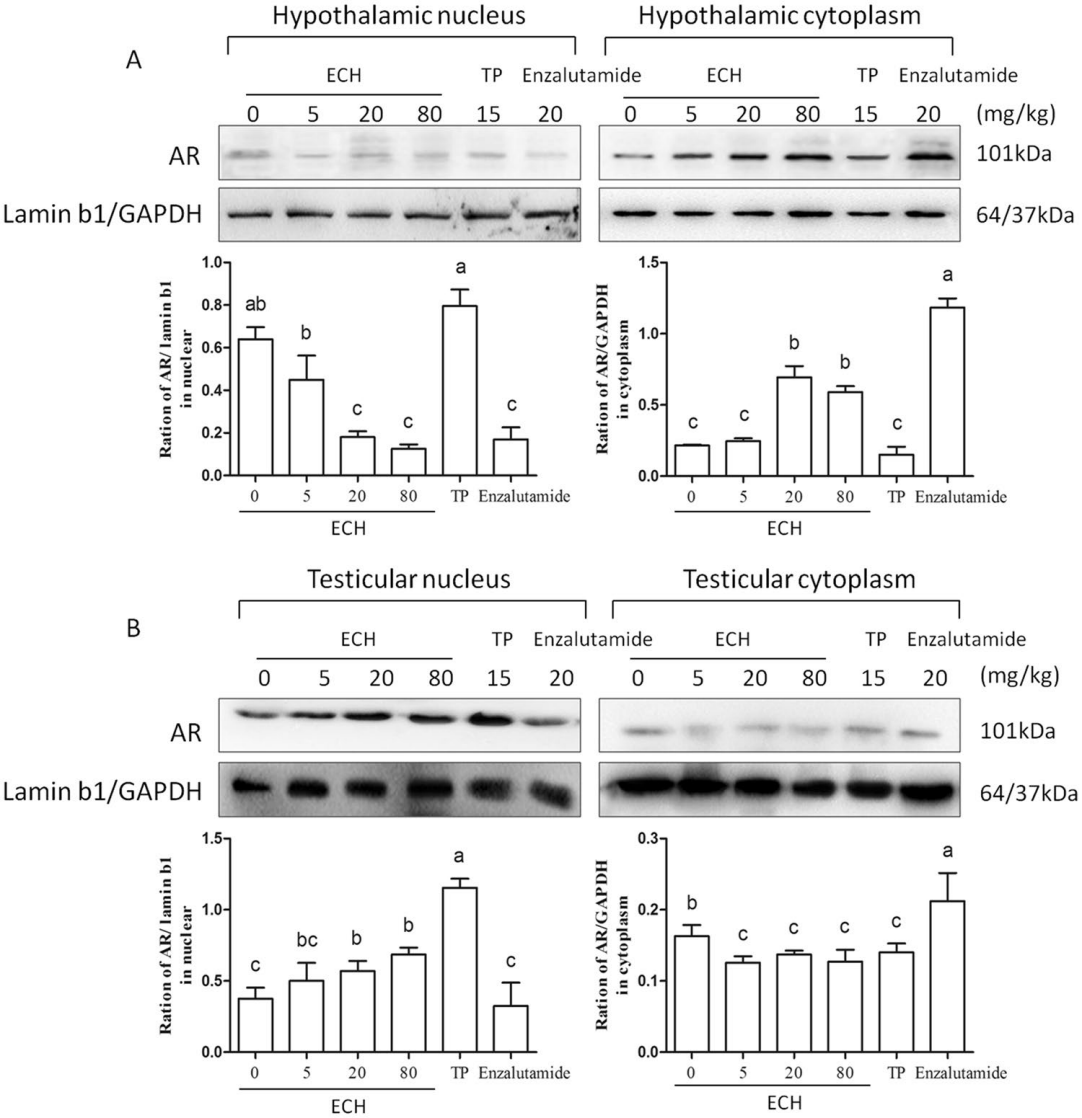
Effect of ECH on AR transportation in testis and hypothalamus. Notes: (**A**) Effect of ECH on AR transport in the hypothalamus. AR protein levels were determined by western blot analysis and bars represent densitometric quantification of nuclear AR normalized to Lamin B and cytoplasmic AR normalized to GAPDH. Each value represents the mean ± SD of six independent experiments. ^a,b,c^Different letters above the bars indicate statistically different groups (*p* < 0.05); (**B**) Effect of ECH on AR transportation in testis.

To test the effect of ECH on the AR translocation, the expression of AR in the cytoplasm and nucleus of testis and hypothalamus was determined using western blot analysis. As shown in Fig. 3A, ECH down-regulated AR protein in hypothalamic nuclei by five-fold compared to the control group. Treatment with enzalutumide, an AR inhibitor, decreased levels of AR protein in hypothalamic nuclei by 4.8-fold. Notably, cytoplasmic AR was higher with ECH treatment and enzalutmide treatment than controls, suggesting that ECH blocks AR transport from the cytoplasm to the nucleus in the hypothalamus. As expected, ECH does not inhibit AR transport from the cytoplasm to the nucleus in testis (Fig. 3B).

### ECH increased the expressions of HPG-axis related genes

ECH significantly increased the expressions of LHβ, LHr, Gnrh 1 and Gnrhr in the mixture pools of encephalon + pituitary, however, there was no significant difference in Gnrh 1 levels after TP treatment. The heatmap analysis shows that the expressions of Gnrh 1 and LH β were highest among HPG-axis related genes.

### AR is a target of ECH

To confirm the relationships between ECH and AR, ECH-Ovalbumin (ECH-OVA) was successfully synthesized (Fig. 5A,B). The molecular weight of ECH-OVA is larger than OVA, so they have a different rates of mobility (Fig. 5A), and the absorbance peak of ECH-OVA is between ECH and OVA (Fig. 5B), suggesting that ECH was successfully conjugated to OVA. Indirect ELISA results (Fig. 5C), show that anti-AR antibody is able to detect AR protein present in wells containing ECH-OVA (indicated by anti-OVA antibody) suggesting that ECH can bind to AR protein.

### ECH combines with AR pocket of Met-894 and Val-713

Compound ECH was docked to the AF2 site on the surface of the human AR, and the theoretical binding mode of ECH and AR was shown in Fig. 5D. Compound ECH adopted a compact conformation binding at the pocket of the human AR. The two phenyl groups of ECH bind at the hydrophobic domain of the AR pocket and maintained close hydrophobic contacts with the residues Leu-712, Val-716, Met-734, Ile-737, Met-894 and Ile-898, whereas the other two sides of ECH were positioned at the entrance of the pocket and made only a few contacts. Detailed analysis showed that both Met-894 (bond length: 2.4 Å) and Val-713 (bond length: 2.7 Å) formed hydrogen bonds with the hydroxy1 groups of the ECH, which was the main interaction between ECH and human AR.

### ECH attenuates BPA-induced reproductive damage

To investigate the effect of ECH on reproductive damage, the sperm quality and levels of sex hormones and steroidogenic enzymes were examined in BPA-induced mice. As shown in Fig. 6, BPA treatment significantly decreased the sperm count and sperm motility by 26.5% and 39.2%, respectively. ECH administration prevented the decrease in sperm count and sperm motility by 35.5% and 30.1%, respectively. BPA administration also resulted in a significant decrease in LH and T secretion, and ECH considerably increased the levels of LH and T by 24.1% and 18.3%, respectively compared to BPA treatment alone (Fig. 6A,B). The mRNA levels of StAR, CYP17A1, 3β-HSD and 17β-HSD in BPA-ECH treatment were significantly increased compared to BPA treatment (Fig. 6C,D).

## Discussion

Ethnopharmacological records and our previous study^13^ confirmed that *Cistanche tubulosa* extract effectively increased sex hormone levels and improved sperm quality. ECH as one of the main compounds in *Cistanche tubulosa*^19,20^, can augment sperm counts and increase the secretion of LH and T (Fig. 1). In this study, we demonstrated that ECH was distributed to the hypothalamus instead of the testes suggesting an indirect effect of ECH on the testes. Indeed, ECH directly inhibited hypothalamic AR activity, AR translocation to the nucleus and HPG-axis related gene expression and also provided protection against BPA-induced reproductive damage.

**Figure 4:**
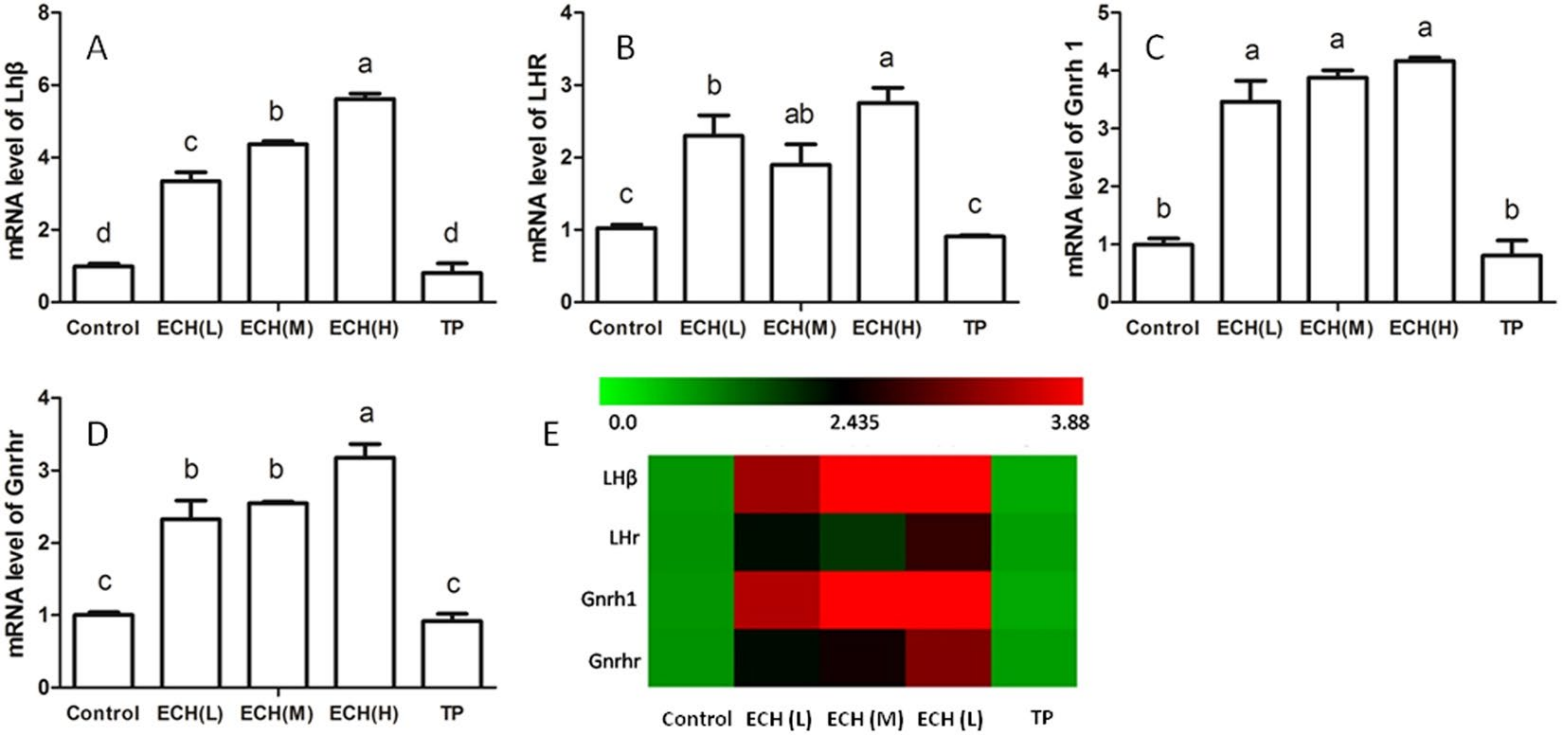
Changes in HPA-related gene expression after ECH treatment. Notes: (**A**–**D**) bars represent the mRNA levels of LHβ, LHr, Gnrh 1 and Gnrhr in encephalon + pituitary. ^a,b,c^Different letters above the bars indicate statistically different groups (*p* < 0.05). (**E**) hierarchical clustering of mRNA expression of different steroidogenic enzymes. Green, green-red and red colors represent low-, medium- and high abundance, respectively, n = 7. Control (treated with normal saline 10 mL/kg), ECH_(L)_ (treated with ECH 5 mg/kg), ECH_(M)_ group (treated with ECH 20 mg/kg), ECH_(H)_ (treated with ECH 80 mg/kg), TP (treated with TP 15 mg/kg).

In studies of human gonadotrophin production, sex hormone levels are tightly regulated by the HPG-axis and a negative feedback loop^21^. Namely, T secretion is markedly increased in response to consistent and sustained increases in blood LH levels in response to a lack of negative androgenic feedback to the pituitary and hypothalamus. In this study, ECH increased the secretion of LH in encephalon + pituitary and production of testosterone in testis and mRNA levels of LHr and Gnrhr in encephalon + pituitary (Fig. 1 and Fig. 4), suggesting that ECH may affect HPG axis negative feedback. Not surprisingly, AR itself plays an important role in the feedback regulation of T and LH levels^22,23^. The male HPG axis paradigm is centered on testosterone providing a negative feedback repression at the level of both the hypothalamus and pituitary gland^24,25^. Previous studies used Foxg1-Cre to ablate genes of interest in the pituitary but not the hypothalamus to determine the relative importance of the two regions. The results demonstrated that AR signaling in the pituitary of male mouse is dispensable in regards to testosterone-dependent regulation of LH secretion^26^. For this reason, we chose to focus on the hypothalamic AR for further study.

**Figure 5:**
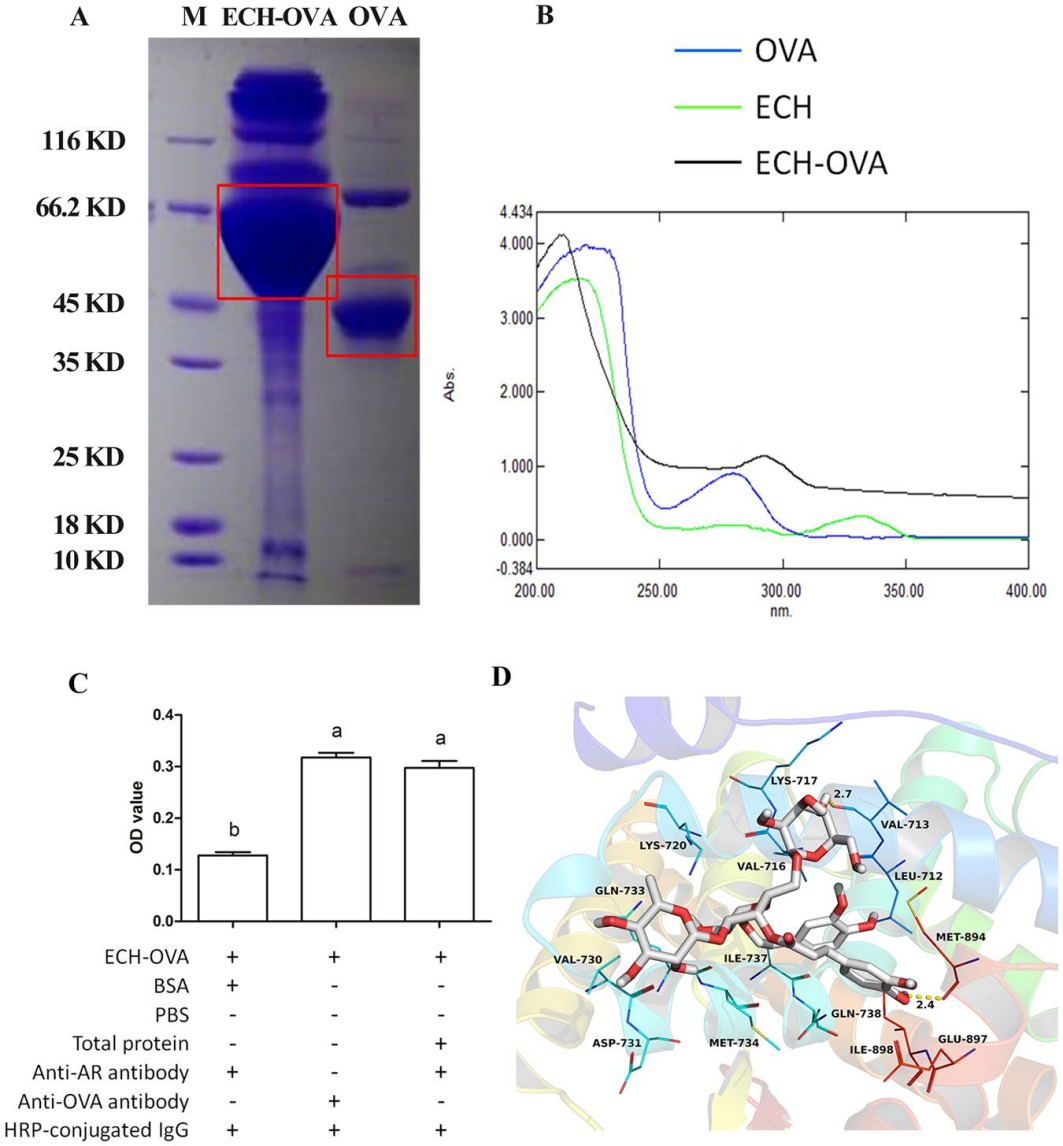
Cytological and in silico investigations of ECH targeting to AR protein. Note: (**A**) SDS-PAGE analysis of ECH-OVA and OVA protein (enclosed by red squares) indicating the molecular weight place of each protein. (**B**) UV absorbance spectra of ECH, ECH-OVA and OVA. The absorption peaks of OVA (blue line), ECH (green line) and ECH-OVA (black line) were 278 nm; the 336 nm and 298 nm, respectively. (**C**) Indirect ELISA assay of the capacity of ECH to bind to AR. BG: blank group; NG: negative group; PG: positive group; EG: experiment group. ^a,b^Different letters above the bars indicate statistically different groups (*p* < 0.05). (**D**) Molecular docking assay for forecasting the binding sites. Both Met-894 (bond length: 2.4 Å) and Val-713 (bond length: 2.7 Å) are the main interactions between ECH and human AR.

As a steroid nuclear hormone receptor, the activity of AR is regulated by the steroid ligand T, the binding of which initiates nuclear translocation and the transcriptional regulatory function of AR^27^. In this study, we showed that ECH treatment inhibited AR translocation from the cytoplasm into nuclei in the hypothalamus, (Fig. 3). Moreover, the PK study showed that ECH can markedly penetrate to the hypothalamus rather than to the testes, suggesting that more ECH crosses the Blood Brain Barrier, possibly via endocytosis, rather than the Blood Testis Barrier. Furthermore, we demonstrated by indirect ELISA the combining capacity of ECH and AR suggesting ECH can directly bind to the AR. This was further supported by results of the molecule docking assay that indicated that putative binding sites for ECH exist at Met-894 and Val-713 in the AR pocket (Fig. 5D). This supports the findings of others that AR participates in the regulation of a negative feed loop and that a reduction in AR-T interactions in the brain results in increased LH secretion^23,28,29^.

Steroidogenesis is a tightly controlled, essential process for the development of spermatogenesis with the involvement of steroidogenic protein (StAR)^30^ and steroidogenic enzymes such as CYP11A1, CYP17A1, 3β-hydroxysteroid dehydrogenase (HSD3β) and 17β-hydroxysteroid dehydrogenase (HSD17β) which convert cholesterol into testosterone^31^. We showed that the mRNA levels of the genes encoding these enzymes significantly increased with ECH treatment whereas the expression of StAR mRNA did not change. This suggests that ECH had no effect on the transfer of cholesterol to the inner membrane of the mitochondria, whereas it may play a role in promoting the cholesterol conversion into testosterone. The conclusion can then be drawn that ECH treatment significantly increases sperm count by augmenting testosterone synthesis and secretion.

**Figure 6:**
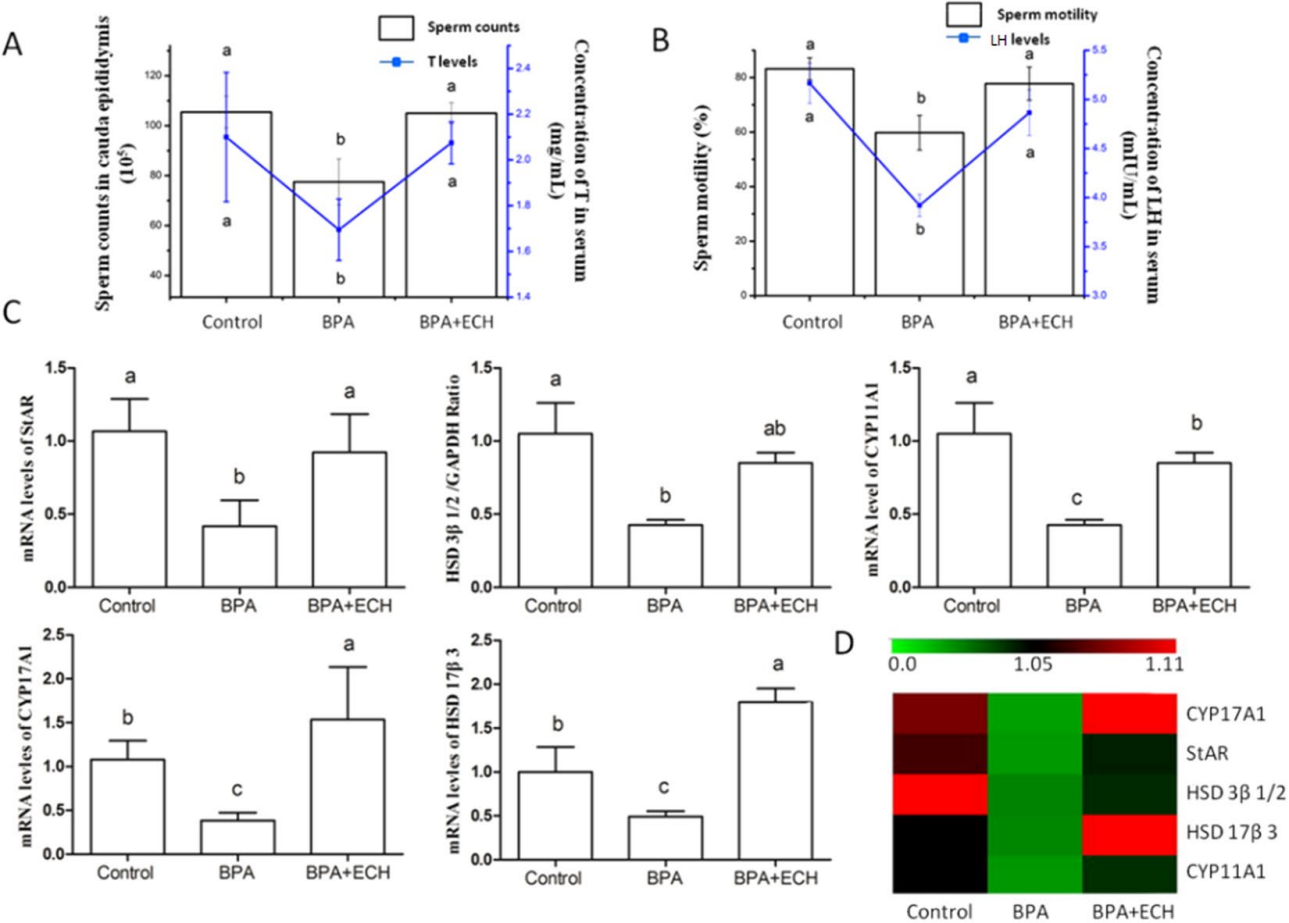
Effect of BPA on ECH-mediated reductions in sperm quantity and quality and mRNA levels of steroidogenic regulatory protein and enzymes. Notes: (**A**,**B**) sperm counts and motility are shown with bars and the concentrations of T and LH are shown with lines. (**C**) The relative mRNA levels of StAR, HSD3β1/2, CYP11A1, CYP17A1 and HSD17β3. ^a,b,c^Different letters indicate statistically different groups (*p* < 0.05). (**D**) Hierarchical clustering of mRNA expression of different steroidogenic enzymes. Green, green-red and red colors represent low-, medium- and high abundance, respectively, n = 7. Control: treated with corn oil 10 mL/kg, bw/d; BPA: treated with BPA 200 mg/kg, bw/d and corn oil; BPA + ECH: treated with BPA 200 mg/kg and ECH 20 mg/kg.

To investigate the protective effect of ECH on sperm injury, bisphenol A (BPA), a typical exogenous endocrine disruptor, was used as a model of male reproductive damage in mice^32^. ECH treatment effectively recovered sperm counts, sperm motility, T and LH levels diminished by BPA (Fig. 6A,B), suggesting that ECH can attenuate sperm injury.

**Figure 7:**
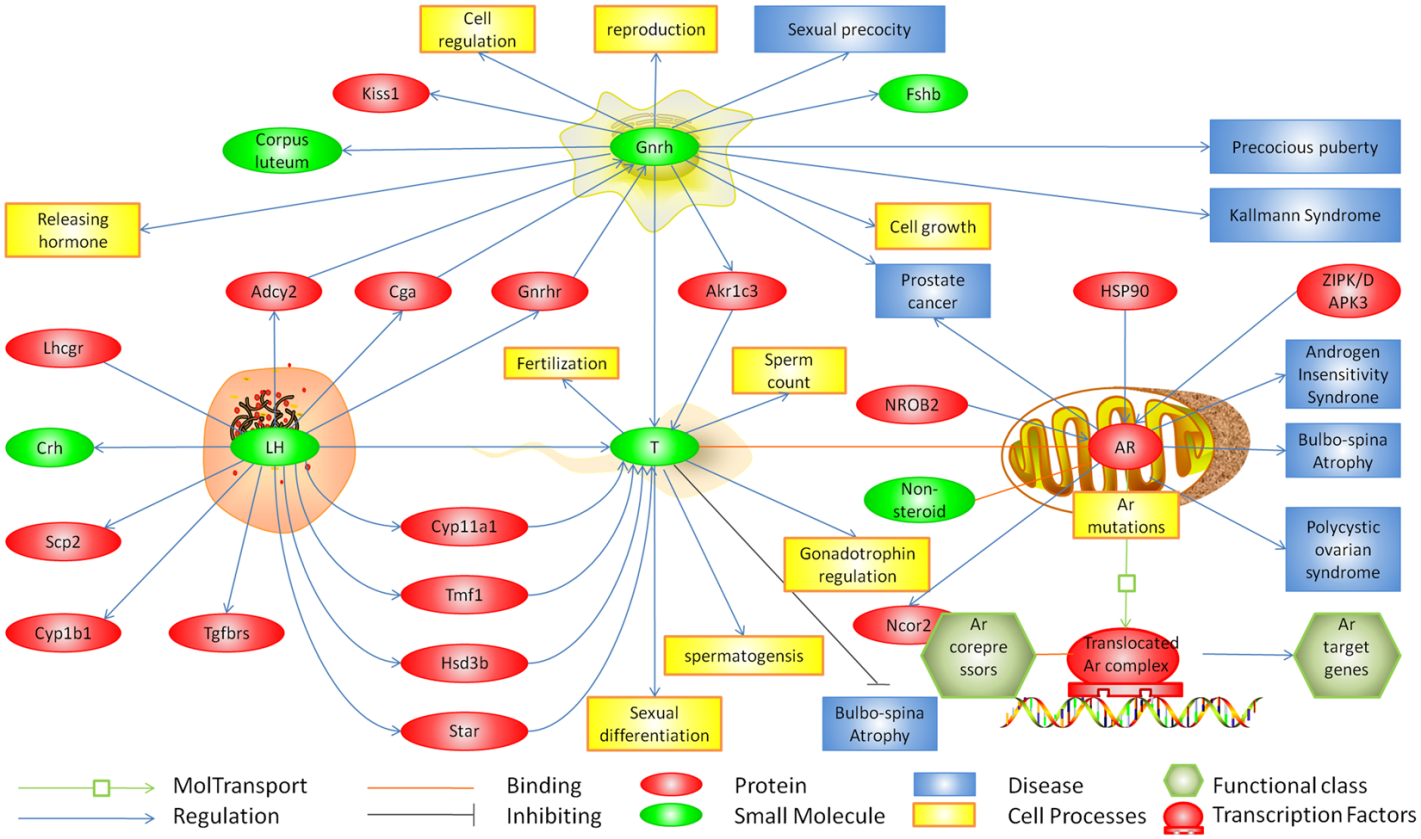
Pathways regulated by selected HPG axis-related hormone and protein in male reproduction as predicted by Pathway Ontology Database and KEGG Pathway Database. Notes: Regulated processes are represented by backgrounds of different colors and shapes, and regulatory events are displayed using arrows and lines. The references linked to each protein were based on Pathway Ontology Database and KEGG Pathway Database, and are not listed.

Compared to the artificially synthesized chemicals for hormone replacement therapy, ECH is a natural non-hormonal compound that affects negative feedback on the HPG axis thereby increasing the secretion of LH, T and sperm quality. This mechanistic pathway may protect the male reproductive system and thus ECH can be considered as a potential natural reproductive protective agent. However, hormone regulations are complex and often lead to systemic effects. As indicated in Fig. 7, ECH increased T and LH secretions, and inhibited AR activation, which may also lead to consequences on other health or disease-associated pathways such as osteoporosis^33^ and depression in men^34^, therefore, the comprehensive effects of ECH should be further investigated.

**Figure 8:**
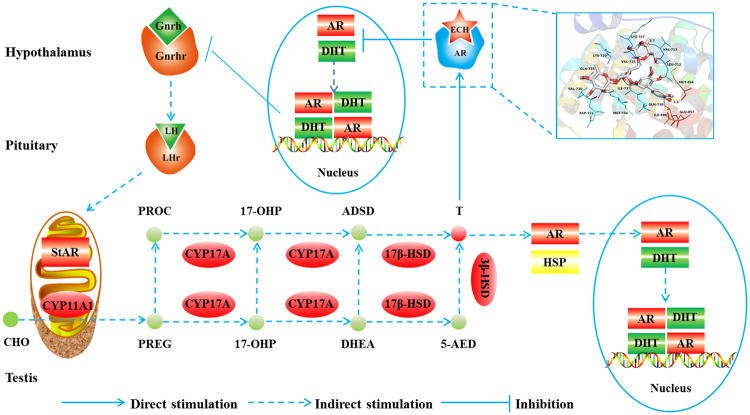
Mechanism by which ECH enhances the sperm counts. Note: ECH targets and inhibits AR transports from the cytoplasm to the nucleus in the hypothalamus, thereby inhibiting the negative feedback of hormones. Positive feedback is subsequently stimulated resulting in increased secretion of Gnrh and LH. LH then stimulates steroidogenic acute regulatory protein (StAR) in conjunction with CYP11A1 in the inner mitochondria membrane of the testis, tethered to the outer mitochondrial membrane. Cholesterol (CHO) in converted, via side-chain cleavage, into pregnenolone (PREG), the hormonal precursor required for synthesis of hormones in the estradiol biosynthesis pathway, including progesterone, dehydroepiandrosterone (DHEA), androstenedione (ADSD), androstenediol (5-AED) and testosterone. Increased testosterone stimulates AR transfer from the cytoplasm to the nucleus in the testis thereby promoting sperm productions.

Taken together, our results indicate that ECH binds to the AR in the hypothalamus and inhibits the transfer of the AR from the cytoplasm to the nucleus. Our data suggest that ECH binds to sites in the AR pocket at amino acids Met-894 and Val-713. This may explain the increase in T and AR levels as a systemic response to balance hormone levels by promoting LH secretion and a subsequent increase in T production and sperm counts (Fig. 8).

## Methods

### Ethics Statement

This study was carried out in strict compliance with the Guidelines for Experimental Animals established by the Ministry of Science and Technology (Beijing, China). All the experimental protocols were reviewed and approved by the Ethics Committee of Anyang Institute of Technology.

### Experimental Animals

Four-week old inbred Kunming mice (22 g–25 g) were obtained from the animal centre of the fourth military medical university (Xi’an, China). The animals were kept under controlled conditions at a temperature of 22 °C ± 2 °C, 70% ± 4% humidity with 12 h light-dark cycling, and free access to feed and water.

### Experiment treatment

#### Experiment 1

Mice were randomly divided into six groups, seven mice in each group (n = 7). ECH (CAS: 82854-37-3; Chengdu Preferred Biotechnology Co., Ltd, Sichuan, China) was administered using an intragastric tube, and testosterone propionate (TP, CAS: 57-85-2; KingYork, Tianjin, China) were given by intramuscular injection once daily for 14 days as per the following experimental design: Control group: normal saline (10 mL/kg), ECH_(L)_ group (5 mg/kg), ECH_(M)_ group (20 mg/kg), ECH_(H)_ group (80 mg/kg), TP (15 mg/kg) and enzalutamide (AR inhibitor, once daily every other day for 14 days; 20 mg/kg; CAS: 915087-33-1; Aladdin, Shanghai, China).

After 2 weeks of treatments, the mice were anesthetized with diethyl ether to collect blood samples for analyses of hormone levels. The mice were then dissected to separate the hypothalamus, encephalon + pituitary, testis and cauda epididymis. Samples of cauda epididymis were put in normal saline with 5% BSA for sperm quality assessment, and the hypothalamus, encephalon + pituitary and testes were frozen in liquid nitrogen and stored at −80 °C for further investigations.

#### Experiment 2

Mice were randomly divided into 10 groups, five animals per group. ECH (20 mg/kg) was administered orally and the mice in each group were anesthetized with diethyl ether at the time points 0.5 h, 1 h, 1.5 h, 2 h, 2.5 h, 3 h, 4 h, 6 h, 9 h and 12 h after administration for sample collection.

Plasma was collected and the heart was exposed. Perfusion of normal saline into the left ventricles was conducted by an infusion apparatus until the liver and lungs were blanched. Then the hypothalamus and testis were collected to determine the tissue concentrations of ECH.

#### Experiment 3

Mice were randomly divided into 4 groups, seven in each group. ECH and BPA (CAS: 80-05-7; Aladdin, Shanghai, China) were administered using an intragastric tube once daily for 6 weeks as per the following experimental design: normal group (corn oil, 10 mL/kg, bw/d), model group: BPA group (BPA 200 mg/kg, bw/d, corn oil), and experimental group: BPA + ECH group (BPA 200 mg/kg; ECH 20 mg/kg).

After 42 days of treatment, the mice were anesthetized with diethyl ether and the blood samples were collected for hormone level analyses. The testis and cauda epididymis were separated and collected. The cauda epididymis was used for sperm quality assessment and the testis were frozen in liquid nitrogen and stored at −80 °C for further investigation.

### Determination of sperm quality

#### Sperm suspension preparation

Cauda epididymides were minced into 5 mL of normal saline with 5% BSA and incubated for 5 min at 37 °C to allow their contents to spread into the medium.

Sperm number: As per the method described by Yokoi (2003), the diluted sperm suspension (1:10; v/v; sperm suspension/10% methanol) was transferred to each counting chamber of a hemocytometer and was allowed to stand for 5 min and then counted under a light microscope (Nikon, Instruments Inc., Japan) at X200 magnification.

Sperm viability: A total of 20 μL of sperm suspension was mixed with an equal volume of eosin-nigrosin stain for 2 min; the un-stained sperms were counted under a light microscope at 200x magnification.

Sperm motility: A total of 10 μL of sperm suspension was putted onto a glass slide and were recorded as either mobile or immobile under a light microscope at 200x magnification.

### Determination of hormone levels

Levels of testosterone (T) and LH were quantified in serum, encephalon + pituitary and testis homogenates using radioimmunoassay (RIA) kits (Beijing Sino-UK Institute of Biological Technology, Beijing, China). Briefly, anti-testosterone or anti-LH IgG antibody (100 μL) and 125-I-conjugated anti-mouse antibody were added to samples or the standard (100 μL), and mixed on a rocker overnight at 4 °C. After washing three times with PBS-Tween 20 (500 μL), the mixtures were centrifuged (3500 rpm/min) at 4 °C for 15 min. The cpm values of the precipitates were assessed by radioimmunoassay instrument (Beijing Sino-western Technology Co. Ltd, CN202M/KZ4GC-1200, Beijing, China) and the concentrations of T and LH were calculated according to the formula of a standard curve. The T and LH coefficient of variation is 2.8% and 3.2% in sample groups, and 2.1% and 2.8% in standard groups, respectively.

### Determination of gene expressions by real-time quantitative PCR

Total RNA was isolated from frozen testicular and encephalon + pituitary tissues using a RNA Simple Total RNA kit (Tiangen, Beijing, China). Quantitative real-time PCR (q RT-PCR) was carried out for the amplification of cDNA using 2 × SYBR Green I PCR Master Mix (Vazyme, Nanjing, China). The PCR procedure consisted of 95 °C for 30 seconds followed by 35 cycles of 95 °C for 15 seconds, 58 °C for 30 seconds and 72 °C for 30 seconds. The melting curve analysis was performed on the PCR products to verify primer specificity and product purity. A dissociation curve was performed for each plate to confirm the production of a single product. The relative abundance of each mRNA was calculated. The PCR primers used for the study are shown in Table 2.

**Table 2:** Primers used for real-time qPCR analyses.

Gene	NCBI reference sequence	Primer sequence (5′–3′)
Gnrhr (Gonadotropin-releasing hormone receptor)	NM_031038.3	F: GCTGCCTGTTCATCATCCCT R: CTGTAGTTTGCGTGGGTCCT
Gnrh1 (Gonadotropin-releasing hormone 1)	NM_012767.2	F: AGGAGCTCTGGAACGTCTGAT R: AGCGTCAATGTCACACTCGG
Lhβ (Luteinizing hormone beta)	NM_012858	F: TGCTGAGCCCAAGTGTGGT R: GGAGGTCACAGGCCATTGG
LHR (Luteinizing hormone receptor)	M81310	F: CTCACCTATCTCCCTGTCAAAGT R: ATGGACTCATTATTCATCCCTTG
StAR (Steroidogenic acute regulatory protein)	NM_031558	F: AGCCAGCAGGAGAATGGAGAT R: CACCTCCAGTCGGAACACCTT
CYP11A1 (Cytochrome P450 11A1)	NM_017286	F: GCAGCGACTCTCTTCTCCTGCG R: GCCATCACCTCTTGGTTTAGGACAATT
CYP17A1 (Cytochrome P450 17A1)	NM_012753	F: GCCACGGGCGACAGAA R: GCCTTTGTTGGGAAAAATCG
HSD3β1/2 (hydroxy-delta-5-steroid dehydrogenase)	NM_001007719	F: GACAGGAGCAGGAGGGTTTGTGG R: CTCCTTCTAACATTGTCACCTTGGCCT
HSD17β3 (hydroxysteroid (17-beta) dehydrogenase 3)	NM_054007	F: AGTGTGTGAGGTTCTCCCGGTACCT R: TACAACATTGAGTCCATGTCTGGCCAG

### Western blot

For AR expression analysis, the extraction and isolation of cytoplasmic and nuclear protein were performed using a Cytoplasmic and Nuclear Protein Extraction Kit (Beyotime, Nanjing, China) according to the manufacturer’s instructions. The concentrations of cytoplasmic and nuclear proteins were assessed using a Bradford Protein Assay Kit (Beyotime, Jiangsu, China). Protein samples (80 μg) were run on a 12% and 5% SDS-PAGE gel and transferred onto PVDF membranes. After blocking, the membranes were incubated with anti-AR IgG antibodies (1:1,000; Bioss; Beijing China), and mouse polyclonal anti-GAPDH antibodies (1:1,000; Wuhan Boster Biological Technology, Wuhan, China) or mouse polyclonal anti-Lamin b1 antibodies for 2 h. The membrane was washed three times with TBST and incubated with an HRP-conjugated rabbit anti-mouse IgG antibody (1:5,000; Bioss; Beijing China). The signal was visualized using the ChemiDoc Imaging System (Tanon-3,500, Shanghai, China).

### High Performance Liquid Chromatography (HPLC) assay

Blood was collected in heparinized glass tubes. The plasma was separated by immediate centrifugation at 6,000 rpm for 10 min and stored at −20 °C for further experiment. The hypothalamus and testis samples from each time point were pooled and homogenized with methanol. After centrifugation at 10,000 rpm at 4 °C for 10 min, the supernatant was concentrated by N_2_ and the residue was dissolved in 50 μL of methanol and filtered through a 0.45 μm filter. Ten μL of the sample filtered liquid was injected into the HPLC system for analysis.

The standard curve consisted of samples containing 50, 100, 250, 500 and 750 ng/mL of the ECH (Chengdu Pufei De Biotech Co., Ltd, Sichuan, China). Plasma quality control samples spiked with 75ng/mL (low), 150 ng/mL (medium) and 300 ng/mL (high) of the ECH was accordingly prepared to measure the accuracy and precision of the method.

Chromatography was performed with an HPLC system (D2000 Elite series, Hitachi, Japan) coupled to an UV detector (L-2400, Hitachi, Japan). Separation was performed on a Thermo-C18 (250 mm × 4.6 mm i.d., 4.6 μm particles) column kept at 25 °C. The mobile phase was a gradient prepared from 0.1% phosphoric acid containing 0.04% trimethylamine (component A) and methanol (component B). The linear gradient was as follows: 70–90% A over 0-2 min, 60–70% A over 2–6 min, 55–60% B over 6–8 min and then returned to 90% A at 8 min immediately. The flow rate was 0.8 mL/min. The UV detector was operated at 332 nm. Peak area was evaluated as the analytical measurement.

### Pharmacokinetic studies in mice

The penetration of ECH to the hypothalamus and testis were subjected to pharmacokinetic analysis with Drug and Statistics software (Drug and Statistics, Mathematical Pharmacology Professional Committee of China). Pharmacokinetic parameters were determined using the non-compartmental method based on statistical moment theory. The preliminary pharmacokinetic parameters including the time to peak constant (*T*_max_), peak concentration (*C*_max_), elimination rate constant (*K*e), elimination half-life (*T*_0.5_), area under curve (AUC_0–12_), apparent distribution volume (*V*c), and clearance (CL) were obtained for further analysis. Brain/plasma ratios were calculated based on AUC_0-t_ values for plasma and brain.

### ECH-Ovalbumin (ECH-OVA) synthesis and identification

A total of 9.8 mg of ECH and 1.0 mg of butanedioic anhydride were dissolved in 2 mL pyridine, mixed for 12 h with stirring at room temperature. The mixture was concentrated by N_2_ and the residue was combined with 10.8 mg of *N*-hydroxysuccinimide (NHS) and 19.3 mg of dicyclohexylcarbodiimide (DCC) and dissolved in 4 mL *N*,*N*-dimethylformamide (DMF) for 12 h with stirring at room temperature. After centrifugation at 2,000 g for 5 min at 4 °C, the supernatant was added to 5 mL of PBS in which 14.4 mg ovalbumin (OVA) was dissolved. The new mixture was stirred for 24 h at 4 °C. The reaction solution was then dialyzed against PBS for three days. The presence of ECH-OVA was confirmed by UV spectra (Shimadzu Scientific Instruments, Inc. Columbia, MD USA) at a wavelength ranging from 190 to 400 nm as well as by denaturing PAGE.

### Indirect ELISA (iELISA)

A microtiter plate was coated with the ECH-OVA (2 μg/100 μL) and incubated overnight at 4 °C. The plate was washed three times with PBST and two times with PBS; and 5% PBSM (PBS containing 5% skimmed milk) was added to block the unbound sites at 37 °C for 2 h. After the plates washed with PBST and PBS, wells were divided into 4 groups; experimental group, 1 μg AR total protein/100 μL; positive group, rabbit anti-OVA antibody (1:1,000); negative group, 1 μg of bovine serum albumin; and a blank group containing PBS. After incubation for 1.5 h, the plates were washed, and 100 μL/well of rabbit HRP-conjugated IgG (1:1,000) was added to the positive group, 100 μL/well of anti-AR was added (1:1,000) to the other groups. After incubation for 1.5 h, rabbit HRP-conjugated IgG (1:5,000) dilution was added to the plates. After incubation at 37 °C for 1 h, the plates were washed three times with PBST and two times with PBS. TMB was then added and incubated for 10 min in the dark at room temperature and 2 M H_2_SO_4_ was added to stop the reaction. The absorbance was read at a wavelength of 450 nm.

### Molecular docking

A molecular docking study was performed to investigate the binding mode of the compound ECH to human androgen receptor (AR) using Autodock vina 1.1.2 (http://vina.scripps.edu). The three-dimensional (3D) structure of AR (PDB ID: 2YHD) was downloaded from Protein Data Bank (http://www.rcsb.org/pdb/home/hone.do). The 3D structure of ECH was obtained by ChemBioDraw Ultra 14.0 and ChemBio 3D Ultra 14.0 software. The AutoDockTools 1.5.6 package (http://mgltools.scripps.edu) was employed to generate the docking input files. The search grid of AR was identified as center x: 36.141, center y: 8.513, and center z: 21.304 with dimensions size x: 15, size y: 15, and size z: 15. The value of exhaustiveness was set to 20. For Vina docking, the default parameters were used if it was not mentioned. The best-scoring pose as judged by the Vina docking score was chosen and visually analyzed using PyMOL 1.7.6 software (http://www.pymol.org).

### Data analysis and statistical methods

The data were analyzed using statistical software SPSS 19.0 (SPSS Inc., Chicago, IL, USA). A one-way ANOVA was employed for comparison among the groups. Tukey’s comparison tests of significant differences among groups were determined. The results were expressed as mean ± standard deviation (SD) using Graph Pad Prism software v.7 (GraphPad Software, Inc, California, USA).

### Ethics approval and consent to participate

Ethical approval for this study was obtained from the Ethics Committee of Anyang Institute of Technology.
